# Prediction of treatment response in rheumatoid arthritis patients using genome‐wide SNP data

**DOI:** 10.1002/gepi.22159

**Published:** 2018-10-12

**Authors:** Svetlana Cherlin, Darren Plant, John C. Taylor, Marco Colombo, Athina Spiliopoulou, Evan Tzanis, Ann W. Morgan, Michael R. Barnes, Paul McKeigue, Jennifer H. Barrett, Costantino Pitzalis, Anne Barton, MATURA Consortium, Heather J. Cordell

**Affiliations:** ^1^ Institute of Genetic Medicine Newcastle University Newcastle upon Tyne UK; ^2^ NIHR Manchester Biomedical Research Centre, Manchester University NHS Foundation Trust Manchester Academic Health Science Centre Manchester UK; ^3^ Leeds Institute of Cancer and Pathology University of Leeds Leeds UK; ^4^ NIHR Leeds Biomedical Research Centre Leeds Teaching Hospitals NHS Trust Leeds UK; ^5^ Centre for Population Health Sciences, Usher Institute of Population Health Sciences and Informatics University of Edinburgh Edinburgh UK; ^6^ Centre for Experimental Medicine and Rheumatology, William Harvey Research Institute, Barts and the London School of Medicine and Dentistry Queen Mary University of London and Barts Health NHS Trust London UK; ^7^ Leeds Institute of Rheumatic and Musculoskeletal Medicine University of Leeds Leeds UK; ^8^ Arthritis Research UK Centre for Genetics and Genomics, Centre for Musculoskeletal Research The University of Manchester Manchester UK

**Keywords:** cross validation, prediction, snp data, treatment response

## Abstract

Although a number of treatments are available for rheumatoid arthritis (RA), each of them shows a significant nonresponse rate in patients. Therefore, predicting a priori the likelihood of treatment response would be of great patient benefit. Here, we conducted a comparison of a variety of statistical methods for predicting three measures of treatment response, between baseline and 3 or 6 months, using genome‐wide SNP data from RA patients available from the MAximising Therapeutic Utility in Rheumatoid Arthritis (MATURA) consortium. Two different treatments and 11 different statistical methods were evaluated. We used 10‐fold cross validation to assess predictive performance, with nested 10‐fold cross validation used to tune the model hyperparameters when required. Overall, we found that SNPs added very little prediction information to that obtained using clinical characteristics only, such as baseline trait value. This observation can be explained by the lack of strong genetic effects and the relatively small sample sizes available; in analysis of simulated and real data, with larger effects and/or larger sample sizes, prediction performance was much improved. Overall, methods that were consistent with the genetic architecture of the trait were able to achieve better predictive ability than methods that were not. For treatment response in RA, methods that assumed a complex underlying genetic architecture achieved slightly better prediction performance than methods that assumed a simplified genetic architecture.

## 1 INTRODUCTION

Rheumatoid arthritis (RA) is an autoimmune disease that results in chronic joint inflammation (McInnes & Schett, 2007). The first choice of treatment of RA is conventional disease modifying anti‐rheumatic drugs (DMARDs) such as methotrexate (MTX; Singh et al., 2016). Patients who do not respond to DMARDs are eligible for biologic or targeted therapies, the most commonly prescribed being tumour necrosis factor α inhibitors (anti‐TNF) therapy (National Institute for Health and Care Excellence (NICE, NICE Technology Appraisal Guidance 375, 2018). Unfortunately, not only does each drug show a significant nonresponse rate in patients (Barrera et al., 2002; Hyrich, Watson, & Symmons, 2006; Soliman et al., 2012), but failure of the treatment may also lead to irreversible joint damage due to uncontrolled inflammation (Smolen et al., 2010). Therefore, it would be of great benefit to be able to predict treatment response, so that patients can be assigned the right treatment at an early stage. Here, we use data from the MAximising Therapeutic Utility for Rheumatoid Arthritis (MATURA) consortium (Barton & Pitzalis, 2017) and focus on predicting the change in C‐reactive protein score (CRP), in 28 swollen joint count score (SJC28) and in erythrocyte sedimentation rate (ESR)—three markers of treatment response—using genome‐wide SNP data in RA patients receiving two different treatments: anti‐TNF and MTX.

A variety of methods have been previously explored for genomic prediction of complex traits. In the human genetics literature, the field has largely been dominated by approaches based on polygenic risk scores (Dudbridge, 2016); however, more advanced approaches derived from either animal breeding or statistical machine learning have also been investigated (Abraham, Kowalczyk, Zobel, & Inouye, 2013; Bermingham et al., 2015; Spiliopoulou et al., 2015; Warren, Casas, Hingorani, Dudbridge, & Whittaker, 2014), including penalised (and related) methods that allow some flexibility in terms of the degree of sparsity (i.e., the number of predictors included in the model) imposed. Most studies have found only small differences in prediction accuracy between sparse and nonsparse methods, although Spiliopoulou et al. (2015) did find that sparse models predicted outcome better in unrelated individuals for traits such as high‐density lipoprotein level (HDL), where there exist SNP effects of moderate size.

A key (but sometimes forgotten) point is the fact that the heritability of a trait imposes an upper limit to the prediction performance that can be achieved using only genetic predictors (Wray et al., 2013). Another sometimes unappreciated point is the fact that large sample sizes are required for building prediction models that attain the theoretically maximum achievable prediction accuracy, which is constrained by the true heritability of the trait (Dudbridge, 2013; Wray et al., 2013). The sample size requirements will be trait‐specific, as they depend on the underlying genetic architecture in terms of the numbers of true genetic effects, as well as on the proportion of variance that each variant explains. For some diseases, a fairly high predictive accuracy (as measured in terms of the area under the curve [AUC]) has been observed with relatively small discovery data sets (Clayton, 2009; Evans, Visscher, & Wray, 2009), but for most complex diseases it has been estimated that discovery sample sizes will need to be in the order of tens, if not hundreds, of thousands (Dudbridge, 2013; Wray et al., 2013) to achieve clinically useful AUCs.

Here, we compare the prediction ability based on a relatively small data set (a few thousand individuals) of 11 methods capable of handling cases where number of SNPs exceeds the number of individuals: lasso, ridge, elastic net, random forests (RF), support vector regression (SVR), sparse partial least squares (SPLS), genome‐wide complex trait analysis (GCTA‐GREML), a Bayesian sparse linear mixed model (BSLMM), a neural network (SkyNet), polygenic risk scores (PRSice), and LD‐based polygenic risk scores (LDpred). We applied each of these methods to predict treatment response in MATURA patients receiving either anti‐TNF or MTX.

Disease Activity Score in 28 joints (DAS28; Felson et al., 1995; Prevoo et al., 1995) is the primary outcome measure used for clinical assessment of disease activity in RA and has been widely validated. It is based on a combination of joint assessments (swelling and tenderness in 28 specified joints) and blood acute phase inflammatory markers including erythrocyte sedimentation rate (ESR) or C‐reactive protein (CRP). Also included is a patient’s visual analogue score of global well‐being (VAS). The individual scores are combined in an algorithm but not equally weighted. Variations of the DAS28, excluding the VAS, for example, have also been validated as measures of treatment response. DAS28 was introduced before the development of imaging‐based diagnostic techniques such as synovitis detection with magnetic resonance imaging (MRI) and ultrasonography (US). There is growing evidence of disparity between the DAS28 and imaging detected synovitis (Brown et al., 2006, 2008; Geng, Han, Deng, & Zhang, 2014; Saleem et al., 2011; Wakefield et al., 2012; Zufferey et al., 2014). However, individual components of the DAS28 such as CRP, ESR, and SJC28 have been found to be associated with imaging‐detected synovitis (Baker et al., 2014; Hensor et al., 2018) suggesting that these markers are the most relevant measures for treatment response. Following this recommendation, we considered change in CRP, SJC28, and ESR as three different measures of treatment response in the MATURA data sets (with ESR available for the MTX cohort only).

As a further illustrative application to a different real data set, we considered a much larger data set available from previous case‐control studies of Primary Biliary Cholangitis (formerly known as Primary Biliary Cirrhosis [PBC]; Mells et al., 2011). Although the biology of PBC does not relate to the biology of RA, we considered this data set to provide an illustrative example of prediction in a real data set that lacks the handicaps of the MATURA data set (small sample sizes and a lack of strong signals). As an additional proof of concept, we also applied the methods to two simulated data sets in which phenotype was simulated based on the real MATURA genotype data: (a) under a sparse model with 22 randomly chosen true causal SNPs; (b) under a polygenic model with 5,000 randomly chosen true causal SNPs.

The remainder of this paper is organised as follows. The Materials section describes the real and the simulated data sets analysed. In the Methods section, we give a brief overview of the statistical methods used. In the Results section, we compare the prediction performance obtained from the different methods, with and without incorporating covariates into the prediction. Finally, we summarise our conclusions in the Discussion section.

## 2 MATERIALS

### 2.1 Anti‐TNF data set

Imputed genotype data at 9,084,265 genome‐wide SNPs for 1,827 patients receiving anti‐TNF treatment were available; this corresponds to essentially the same data set described by Massey et al. (2018). We performed quality control (QC) on the imputed SNP data using standard procedures outlined in Anderson et al. (2010). Individuals were excluded if the reported sex did not match the sex assessed by genotype, and also for elevated missingness rate, outlying heterozygosity rate, outlying ethnicity and relatedness. SNPs were excluded if they had a post‐imputation INFO score < 0.8. Genotype hard calls were set to missing if the posterior probability was <0.9. The data was filtered by minor allele frequency (MAF; >0.01), Hardy–Weinberg disequilibrium (*p* > 0.000001) and missing genotype rate (<0.05). The SNP genotypes were encoded according to the number of copies of the minor allele possessed. The post‐QC data set was comprised of 1,819 individuals and 4,542,023 SNPs.

For analysing the change in CRP, we defined the phenotype as the difference between the follow‐up CRP measure (measured at 6 months, or 3 months if this was not available) and the baseline CRP measure on the log scale, that is, $\log\left({\text{CRP}}_{fu}\right)-\log\left({\text{CRP}}_{bl}\right)$. We adjusted the phenotype for the log baseline measure, the drug type (Infliximab, Etanercept, Adalimumab, Certolizumab pegol, Golimumab) and the first 10 principal components (PCs) of the SNP genotypes (to account for population stratification) using linear regression. We took these standardised residuals as our final CRP phenotype. The CRP phenotype was available for 1,088 individuals, out of which 972 individuals had a 6 months follow‐up measure, and 116 individuals had a 3 months follow‐up measure.

For analysing the change in SJC28, the difference between the follow‐up SCJ28 measure (${\text{SJC28}}_{fu}$; measured at 6 months, or 3 months if this was not available) and the baseline SJC28 measure (${\text{SJC28}}_{bl}$) was adjusted for the baseline measure, the drug type, the first ten PCs and a binary indication of whether or not patients received another disease‐modifying anti‐rheumatic drug (DMARD) in addition to the anti‐TNF treatment. (This covariate was also considered but not found to be significant for modelling the change in log CRP, above). The standardised residuals were taken as the SJC28 final phenotype. The SJC28 phenotype was available for 1,782 individuals, out of which 1,638 individuals had a 6 months follow‐up measure and 144 individuals had a 3 months follow‐up measure.

For analysing the change in ESR, we defined the phenotype similarly to CRP, that is, $\log\left({\text{ESR}}_{fu}\right)-\log\left({\text{ESR}}_{bl}\right)$. It was then adjusted for the baseline measure, the drug type, the DMARD indicator, gender and the first 10 PCs, with the standardised residuals taken as the final phenotype. (Gender was also considered but not found to be significant for modelling the change in log CRP or SJC28, above). The ESR phenotype was available for 1,575 individuals, out of which 1,462 individuals had a 6 months follow‐up measure and 113 individuals had a 3 months follow‐up measure.

### 2.2 MTX data set

Imputed genotype data at 7,542,957 genome‐wide SNPs for 828 patients receiving MTX treatment (collected across a variety of cohorts, see Taylor et al., 2018 for details) were available; this corresponds to the MATURA‐owned data on a subset of the patients described by Taylor et al. (2018). Individual and SNP QC (as described above for the anti‐TNF data set) resulted in a data set with 657 patients and 6,291,430 SNPs.

For analysing the change in CRP, the phenotype was defined as $\log\left({\text{CRP}}_{fu}+1\right)-\log\left({\text{CRP}}_{bl}+1\right)$, and was adjusted for $\log\left({\text{CRP}}_{bl}+1\right)$, the cohort effect and the first 10 PCs. (The reason for adding 1 to the argument of the log function was to make the argument positive for cases where CRP = 0; this issue did not occur for the anti‐TNF data set described above). The standardised residuals were then taken as the final CRP phenotype. The CRP phenotype was available for 618 individuals.

For analysing the change in SJC28, the phenotype was defined as $\sqrt{{\text{SJC28}}_{fu}}-\sqrt{{\text{SJC28}}_{bl}}$ and was adjusted for $\sqrt{{\text{SJC28}}_{bl}}$, the cohort effect and the first 10 PCs. The motivation for taking the square root was to achieve a more normally distributed trait value; this transformation was not found to be required in the anti‐TNF data set described above. The standardised residuals were taken as the final SJC28 phenotype that was available for 629 individuals. For both CRP and SJC28, the follow‐up measurement was taken 3–6 months after initiating the MTX treatment, although precise duration of treatment was not available.

### 2.3 PBC data set

PBC is an autoimmune liver disease for which a number of genome‐wide significant loci have previously been found (Cordell et al., 2015; Mells et al., 2011). Here, we utilised post‐QC genome‐wide SNP data available from the case‐control study of Mells et al. (2011), comprising 501,358 SNPs measured in 6,977 individuals (1,816 PBC cases and 5,161 controls).

### 2.4 Simulated data set (sparse model)

Phenotype data for a hypothetical quantitative trait were simulated using the real genotype data from the anti‐TNF (CRP) data set. We started by selecting 22 randomly chosen causal SNPs (one SNP per chromosome). Simulation of the phenotype was performed using the GCTA‐GREML software (https://cnsgenomics.com/software/gcta/) (Yang, Lee, Goddard, & Visscher, 2011), with SNP effects simulated from $N\left(0,0.05^{2}\right)$ and the overall heritability parameter set to $h^{2}=0.8$. This relatively large value of heritability was chosen to simulate strong signals in the data. We refer to this data set as SimSparse.

### 2.5 Simulated data set (polygenic model)

Phenotype data for a hypothetical quantitative trait were simulated using the real genotype data from the anti‐TNF (CRP) data set. Here, we used 5,000 randomly chosen causal SNPs. Simulation of the phenotype was performed using the GCTA‐GREML software (Yang et al., 2011), with SNP effects simulated from $N\left(0,0.05^{2}\right)$ and the overall heritability parameter set to $h^{2}=0.8$, similar to the SimSparse data set. We refer to this data set as SimPoly.

## 3 METHODS

We investigated 11 methods capable of handling cases where number of SNPs exceeds the number of individuals: lasso, ridge, elastic net, random forests (RF), support vector regression (SVR), sparse partial least squares (SPLS), genome‐wide complex trait analysis (GCTA‐GREML), a Bayesian sparse linear mixed model (BSLMM), a neural network (SkyNet, polygenic risk scores (PRSice), and LD‐based polygenic risk scores (LDpred)). We used mean imputation (expected dosage value) of SNP genotypes when using methods that do not allow missing genotypes. Lasso (Tibshirani, 1996), ridge (Cessie & Houwelingen, 1992), and elastic net (Zou & Hastie, 2005) are penalised regression approaches that induce different amounts of sparsity, depending on the type of penalty used. SPLS (Chun & Keleş, 2010) is also a sparse method that utilises latent component decomposition to reduce dimensionality. GCTA‐GREML (Yang et al., 2011) is a nonpenalised approach that implements linear mixed model analysis, where the effects of SNPs are modelled as random effects, with the covariance matrix of the cumulative genetic effect being proportional to the genetic relationship matrix (GRM) between individuals. BSLMM (Zhou, Carbonetto, & Stephens, 2013) is a hybrid approach of a linear mixed model and a sparse regression, where sparsity is applied to the fixed effects. RF (Breiman, 2001) involves generating a collection of tree‐structured predictors where each node of a tree is split using the best among a subset of predictors randomly chosen at that node. The final prediction is made based on the mean prediction over the individual trees. SVR (Vapnik, 1995) is a nonparametric kernel‐based technique whose aim is to learn a nonlinear loss function by mapping into high dimensional kernel induced feature space. Here, we apply the ϵ‐SVR model with a nonlinear kernel function (Long, Gianola, Rosa, & Weigel, 2011), which has a sparse solution and allows nonlinear relationships between the SNPs and the phenotype. SkyNet (Graff, Feroz, Hobson, & Lasenby, 2014) is an implementation of artificial neural networks which are used to represent nonlinear relationships between a set of inputs and outputs. By learning a mapping between the inputs and the outputs, given a set of training data, one can make predictions of the outputs for new input data. PRSice (Euesden, Lewis & O’Reilly, 2015) is a polygenic risk score method that calculates the best‐fit polygenic risk scores from a number of *p*‐value thresholds. LDpred (Vilhjálmsson et al., 2015) is a polygenic risk score method that acconts for linkage disequilibrium (LD) between the SNPs. For a more detailed description of each of these methods, please see the Appendix.

We assessed the prediction accuracy of the different methods through a variety of different measures: (a) the Pearson correlation coefficient between observed and predicted trait values, (b) the calibration slope (the slope of the best fit line when plotting predicted trait values on the *x*‐axis against observed values on the *y*‐axis; a slope of 1 suggests perfect calibration (Piñeiro, Perelman, Guerschman, & Paruelo, 2008; Steyerberg et al., 2010), and (c) prediction mean squared error (PMSE), which is the average squared difference between observed and predicted trait values (lower values indicate better fit). We note that although for the binary oucome (PBC data set), assessing correlation, slope and PMSE is less natural, they are still well defined quantities and we use them for general comparison. Predictive perfomance was assessed through 10‐fold cross validation, with nested 10‐fold cross validation used to tune the model hyperparameters when required. In 10‐fold cross validation, 1/10 of the data are held out to be used as a test data set, with the other 9/10 of the data used to fit (estimate) the prediction model. The procedure is then repeated 10 times with different 1/10 of the data being held out, such that all the data is ultimately used as testing data.

The first step within each fold involved reducing the number of the SNPs using an LD‐based clumping procedure implemented in the PLINK software (http://zzz.bwh.harvard.edu/plink/) (Purcell et al., 2007). A similar “supervised feature selection” procedure (which is designed to select a reduced number of SNPs with larger effects for input into the main analysis, while allowing for the LD between SNPs) was used by Bermingham et al. (2015). LD‐based clumping (‐‐clump command) utilises GWAS results to clump SNPs based on their LD with the SNPs nearby and the *p*‐values. Clumps are formed around SNPs with pre‐specified *p*‐values (‐‐clump‐p1 and ‐‐clump‐p2 parameters specify the *p*‐value threshold for the index SNP and the clumped SNPs, respectively), and the index SNPs are then used to represent all the SNPs in a clump. Table 1 shows the reduced number of SNPs used for each method. To reduce the number of SNPs to approximately 40,000–42,000, we used ‐‐clump‐p1 0.05 ‐‐clump‐p2 0.05; to reduce the number of SNPs to approximately 9,000–10,000, we used ‐‐clump‐p1 0.01 ‐‐clump‐p2 0.01; to reduce the number of SNPs to approximately 340,000–370,000, we used ‐‐clump‐p1 0.5 ‐‐clump‐p2 0.5. Additional parameters for the LD‐clumping are ‐‐clump‐kb that specifies the physical distance threshold for clumping, and ‐‐clump‐r2 that specifies the LD threshold for clumping. In our analysis, we used ‐‐clump‐kb 100 ‐‐clump‐r2 0.8. For PRSice, the LD‐clumping is performed as a default built‐in option of the software with parameters ‐‐clump‐p1 1.0 ‐‐clump‐p2 1.0 ‐‐clump‐kb 250 ‐‐clump‐r2 0.1, resulting in 140,000–170,000 SNPs.

**Table 1:** The number of SNPs included in the analysis and the tuning parameters that require cross validation, for the MATURA data sets, for the 11 methods.

Method	Number of SNPs for anti‐TNF cohort	Number of SNPs for MTX cohort	Tuning parameters that require cross validation
Lasso			
Elastic Net	40,000	42,000	Penalty parameter
Ridge			
RF	9,000	9,000	Number of variables to split
SVR	9,000	9,000	Standard deviation for Gaussian RBF kernel
SPLS	40,000	42,000	Number of components, sparsity parameter
GCTA‐GREML	4,542,024	6,291,430	NA
PRSice*			
BSLMM	40,000	42,000	NA
SkyNet	9,000	9,000	NA
LDpred	340,000	370,000	NA

*Note.* The prediction is based on the SNP effects only. For the PBC data set, we use SVM (Support Vector Machine) instead of SVR on account of binary outcome.

* For PRSice, the LD‐clumping is performed within the software, resulting in ≈ 140,000 SNPs for the anti‐TNF cohort, and ≈ 170, SNPs for the MTX cohort.

To estimate heritability for the RA data sets considered here, we used the LDAK method (Speed & Cai, 2011; Speed, Hemani, Johnson, & Balding, 2012), which uses a modified kinship matrix in which SNPs are weighted according to their LD with SNPs nearby, MAF and imputation accuracy. For comparison, we also applied a variety of other methods for heritability estimation (Supporting Information Table I).

To simplify the calculations, the adjustment for covariates was not done fold‐wise, but rather before the division of the data sets into cross validation folds. This “global” covariate adjustment could in theory cause a slight contamination of the testing subsets with information from the training subsets, resulting in an over‐optimistic assessment of prediction performance. However, we anticipated that the amount of contamination should be small and would not substantially change the overall prediction results. This intuition was borne out by our results from a limited evaluation in which covariate adjustment was carried out within each fold rather than globally, as demonstrated later (see Section 4.2).

We note that the methods that we investigated have different computational requirements. The cross validation procedures were programmed in R version 3.4.1 and were executed fold‐wise. For all methods but BSLMM and SkyNet, the discovery stage of the analysis of each fold took between 5 min and a couple of hours on a SLURM‐based (Yoo et al., 2003) batch‐queuing cluster running in an OpenStack environment (The OpenStack project, 2018). (The cluster consisted of two identical 23‐core virtual machines with 64 GB RAM and 100 GB disk). However, BSLMM and SkyNet required longer times: depending on the data set, SkyNet took between 1 and 6 days to construct an ANN, while BSLMM took between 10 and 15 days to generate 110,000,000 Markov chain Monte Carlo (MCMC) iterations. Nevertheless, for some parameters of BSLMM, the MCMC chain obtained by generating 100,000,000 iterations with default tuning parameters, after discarding 10,000,000 iterations and thinning by 10,000 iterations, still showed a lack of convergence, indicating that careful tuning and/or longer run times are required (Supporting Information Figure 1).

## 4 RESULTS

### 4.1 Prediction based on SNPs alone

**Figure 1:**
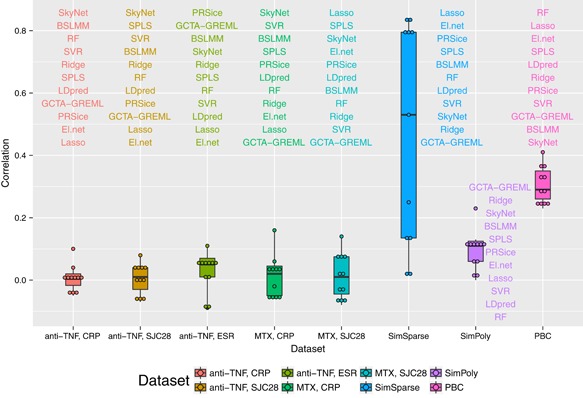
Pearson correlation coefficient from the prediction analyses for the 11 methods for all the data sets [Color figure can be viewed at wileyonlinelibrary.com]

**Figure 2:**
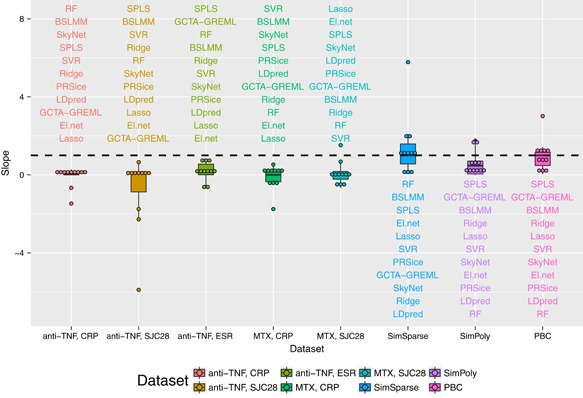
Calibration slope (a slope of 1 suggests perfect calibration) from the prediction analyses for the 11 methods for all the data sets [Color figure can be viewed at wileyonlinelibrary.com]

**Figure 3:**
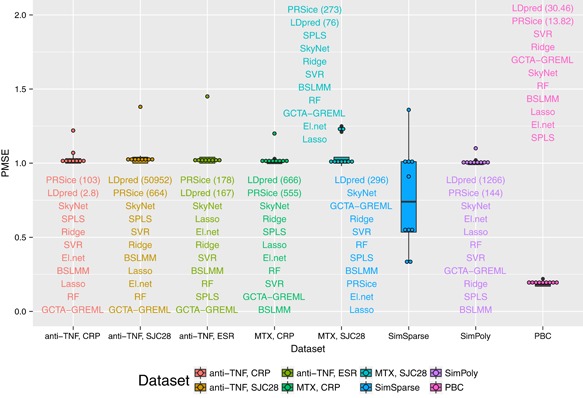
Prediction mean squared error (PMSE; lower values indicate better fit) from the prediction analyses for the 11 methods for all the data sets [Color figure can be viewed at wileyonlinelibrary.com]

**Figure 4:**
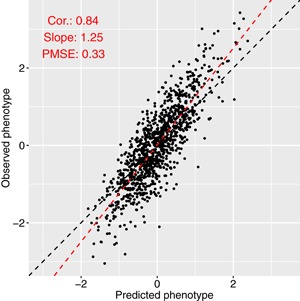
Prediction with lasso for the SimSparse data set. The black dashed line is the equality line; the red dashed line is the best fit line [Color figure can be viewed at wileyonlinelibrary.com]

The values of the Pearson correlation coefficient, the calibration slope, and the PMSE from the compared methods are presented in Figures 1, 2, and 3. For the data simulated under a sparse model (SimSparse), the sparse methods such as lasso, elastic net, SPLS and BSLMM achieve better prediction than the other methods. This result is as expected because the data were simulated according to a sparse model, and so models that do not violate the assumption of the data generating mechanism perform better than models that do. Specifically, lasso (see Figure 4) shows the best prediction, followed by elastic net. This is consistent with the fact that the data were simulated using a few causal SNPs that show strong association with the phenotype, as illustrated by the Manhattan plot obtained from this data set when analysing each SNP individually via linear regression (Supporting Information Figure 2g). SPLS and BSLMM were outperformed by lasso and elastic net, which may be explained by the fact that the two former methods employ sparsity mechanisms that are unnecessarily complicated for data generated according to a simpler sparse model. On the other hand, polygenic models such as ridge regression and GCTA‐GREML perform worse on the SimSparse data set, consistent with the fact that the data were not simulated under a polygenic model. To investigate further the reason behind the poor prediction ability of polygenic methods on the SimSparse data set, we reduced the genome‐wide SNP data to 22 genomic regions (±5 MB around each true causal SNP; the reduced data set was comprised of 71,835 SNPs) and analysed the resulting data with GCTA‐GREML. Even though the calibration slope remained unchanged (0.94), the prediction ability greatly improved in terms of the correlation (0.27 vs 0.02 previously) and the PMSE (0.99 vs 1.04 previously) as illustrated in Supporting Information Figure 3. This suggests that, when the data generating mechanism is sparse, polygenic methods such as GCTA‐GREML can benefit from reducing the genome‐wide data to candidate regions.

For the data simulated under a polygenic model (SimPoly), GCTA‐GREML achieved better prediction than the other methods, followed by ridge regression, which is again consistent with data generating mechanism. However, out of 10 cross validation folds, convergence was not achieved for 4‐folds, therefore the prediction results are based on 6‐folds only. In general, the prediction for the SimPoly data set is worse than that for the SimSparse data set, which can be explained by the fact that in a polygenic architecture each individual signal is rather weak (Supporting Information Figure 2h), despite the large true value of the heritability.

For the real MATURA data, all methods show poor prediction in general (small correlations, large PMSEs, and slopes that are far from 1). Nevertheless, some methods perform better than the others across the data sets. For example, while SkyNet and SPLS achieve a positive but small correlation across all MATURA data sets (the correlation ranges from 0.07 to 0.16 for SkyNet, and from 0.04 to 0.08 for SPLS), lasso and elastic net do not show consistency in the direction of the correlation across the data sets. Specifically, lasso and elastic net achieve positive correlation only for the MTX SJC28 data set. This may reflect the fact that this trait shows one reasonably compelling signal of association, as supported by the Manhattan plot of the associations between the SNPs and the phenotype (Supporting Information Figure 2e). However, this association signal was not maintained when adding in the additional cohorts considered by Taylor et al. (2018), and so most likely represents a statistical false positive. As for the other traits, the Manhattan plots of the *p*‐values show little in the way of significant associations between the SNPs and the phenotype (Supporting Information Figure 2a–d). This suggests that any genetic effects that exist for these traits operate via a polygenic architecture, which is not accounted for by simple sparse models. While we might expect methods that assume a polygenic architecture (such as Ridge, GCTA‐GREML, and BSLMM) to better model the genetic architecture of these traits, only BSLMM consistently performs better than the other methods (with correlation ranging between 0.04 and 0.07). This suggests that the true genetic architecture of these traits may be rather complex and so better explained by a model that accommodates different types of effects.

We hypothesised that the existence of stronger genetic effects and larger sample sizes should improve the prediction performance in real data. To investigate this hypothesis, we analysed the PBC data set which shows a number of significant associations between the SNPs and the phenotype based on the Manhattan plot of the *p*‐values (Supporting Information Figure 2f). The results presented in Figures 1–3 indicate much better prediction performance for the PBC data set than for the MATURA data sets. For the PBC data set, the correlation ranges between 0.23 and 0.41 across different methods, the slopes are close to the values achieved for the simulated data, and the PMSEs do not exceed the phenotypic variance (0.19) for most methods.

**Figure 5:**
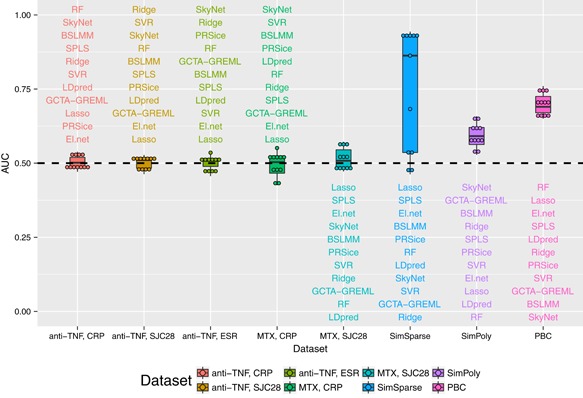
Area under the curve (AUC) from the prediction analyses for the 11 methods for all the data sets, after transforming the phenotype to a binary format [Color figure can be viewed at wileyonlinelibrary.com]

In spite of the positive results found with the PBC data set and with the SimSparse data set, we note that the predictive performance achieved even in these best‐case scenarios does not provide very precise prediction of trait values. However, it is arguable whether precise prediction of the trait values is in fact the most relevant goal for clinical purposes; it would be perhaps more useful simply to be able to predict whether an individual will be a responder or a nonresponder. We therefore transformed the observed and predicted outcomes into a binary format (responder/nonresponders). Our transformation was guided by the EULAR‐response criteria (EULAR response criteria, 2018; van Gestel & Prevoo, 1995), which define good responders according to the improvement in the DAS28. We note that while the EULAR‐response criteria define three response categories (good/moderate/poor), we define only two response categories (responder/nonresponder), with response corresponding to the improvement of at least 0.6 units in the DAS28 score. Following the recommendations of Hensor et al. (2018) (who found that, out of the individual components of the DAS28, it is CRP and SJC28 that are the most predictive of imaging‐detected synovitis) and of Massey et al. (2018) (who found that only ESR and SJC28 are highly heritable), we based our transformation of CRP, SJC28, and ESR on their contribution to the DAS28. We therefore defined individuals as responders if $\log\left({\text{CRP}}_{bl}\right)-\log\left({\text{CRP}}_{fu}\right)>1.67$ for the CRP phenotype; if $\sqrt{{\text{SJC28}}_{bl}}-\sqrt{{\text{SJC28}}_{fu}}>2.14$ for the SJC28 phenotype; if $\log\left({\text{ESR}}_{bl}\right)-\log\left({\text{ESR}}_{fu}\right)>0.857$ for the ESR phenotype (see Table 2 for the number of responders and nonresponders in the MATURA data sets). The values of the area under the curve (AUC) presented in Figure 5 suggest that for the MATURA data sets, the prediction ability is only rarely slightly better than that of a random guess, with AUC ranging from 0.43 to 0.57, consistent with the relatively poor prediction results achieved for the original quantitative phenotypes. In comparison, ROC curves for the PBC data show better predictive ability, which is illustrated by AUCs ranging from 0.65 to 0.76. Also, the SimSparse data set achieves higher AUC values with sparse methods such as lasso, while the SimPoly data set achieves better prediction with polygenic methods such as GCTA‐GREML, in accord with the prediction achieved for the quantitative phenotypes.

**Table 2:** The number of responders and nonresponders after the transformation of the phenotype to the binary format for the MATURA data sets

Treatment	Phenotype	Responders	Nonresponders	Total
Anti‐TNF	CRP	192	896	1,088
	SJC28	660	1,122	1,782
	ESR	513	1,062	1,575
MTX	CRP	144	474	618
	SJC28	161	468	629

With respect to overall trait heritability, the heritabilities estimated with LDAK for the phenotypes considered in the anti‐TNF cohort (relating to change in CRP, SJC28, and ESR) were 0.024 (SE 0.378) for CRP, 0.255 (SE 0.232) for SJC28 and 0.534 (SE 0.269) for ESR. The estimated heritabilities for the phenotypes considered in the MTX cohort (relating to change in CRP and SJC28) were 0.187 (SE 0.678) for CRP and 0 (SE 0.657) for SJC28. Large standard errors of the estimates indicate very low precision and can be explained by small sample sizes. We obtained somewhat different results with a variety of other methods for heritability estimation (Supporting Information Table I), in accord with previous studies (Mirkov et al., 2015; Speed & Cai, 2011). However, all the methods estimated heritability with rather low precision, consistent with suggestion (Yang, Zeng, Goddard, Wray, & Visscher, 2017) that sample sizes in the order of tens of thousands are needed to obtain high precision of heritability estimates.

### 4.2 Prediction based on SNPs and covariates

To investigate the contribution of SNPs to overall prediction, for three selected methods (lasso, BSLMM, and SkyNet) we re‐analysed the anti‐TNF data set (CRP and SJC28 phenotypes) while additionally considering the contribution to prediction achieved by the covariates. The covariates used were the baseline trait measure, the drug type and the first 10 PCs of the SNP genotypes (for the SJC28, the covariates also included the DMARD indicator). Since all three methods operate by default on standardised residuals, the standardised residuals used for model‐building were here obtained within each cross validation fold, after fold‐wise adjustment for the covariates. Following model building, we then back‐transformed the predicted standardised residuals in the held‐out portion of the data to the original phenotype scale (using the estimated regression coefficients for the covariates obtained from the training portion of the data), to generate predicted phenotypes (predicted on the basis of both SNPs and covariates) that could be used for comparison with the observed phenotypes.

For each data set, the correlation, the calibration slope, the PMSE, and the AUC from the three methods are presented in Table 3. Higher correlations and AUCs, and slope values closer to 1, in comparison to the analysis based on SNPs only, indicate better prediction. The PMSEs in this analysis and in the analysis based on SNPs alone (Figures 1–3) are not directly comparable because the final phenotype is standardised in the analysis based on SNPs alone and is not standardised in this analysis. However, an improvement in the PMSEs is indicated by the fact that the PMSE values are substantially smaller than the phenotypic variances (which are 4.97 for the CRP data set and 141.482 for the SJC28 data set), while in the analysis based on SNPs alone, the PMSE values are very close, and sometimes even larger than, 1 (the variance of standardised phenotype). The AUC values for the binary transformation (ranging from 0.71 to 0.81), also indicate reasonably good predictive ability.

**Table 3:** Pearson correlation coefficient (Cor.), the calibration slope (a slope of 1 suggests perfect calibration), the prediction mean squared error (PMSE; lower values indicate better fit), and area under the curve (AUC) for the anti‐TNF data sets, for the three methods

Data Set	Method	Cor.	Slope	PMSE	AUC
Anti‐TNF (CRP)	Lasso	0.44	0.98	1.27	0.81
	BSLMM	0.43	0.91	1.29	0.8
	SkyNet	0.34	0.5	1.57	0.74
Anti‐TNF (SJC28)	Lasso	0.77	0.99	17.63	0.73
	BSLMM	0.76	0.97	18.7	0.73
	SkyNet	0.7	0.81	23.45	0.71

*Note*. The prediction is based on the SNP effects and the covariates.

To investigate the improvement in the prediction after including the covariates in the analyses, we analysed the anti‐TNF, CRP data set while using (a) a linear regression model with nongenetic covariates as predictors (the baseline trait measure and the drug type), (b) a linear regression model with genetic (first 10 PCs of the SNP genotypes) and nongenetic (the baseline trait measure and the drug type) covariates as predictors, (c) lasso using SNPs and also genetic and nongenetic covariates as predictors (the same covariates as in (a) and (b)). All three methods achieved a (reasonable) identical prediction accuracy as measured by the correlation (0.44), the slope (0.98) and the PMSE (1.27; Supporting Information Figure 4). This indicates that most of the information about prediction comes from the nongenetic predictors, supporting the conclusions (in relation to prediction of change in DAS28) from a previous anti‐TNF study (Sieberts et al., 2016).

## 5 DISCUSSION

Here, we have presented a comparison of 11 methods for predicting the treatment response in RA patients using genome‐wide SNP data. We showed that the SNP data contribute very little information to the prediction achieved using clinical covariates only, in accord with previous studies of cardiovascular disease (Morris et al., 2016) as well as of treatment response in RA (Sieberts et al., 2016). This can be explained by the fact that the SNPs show no strong association signals, as is evident from the Manhattan plots of *p*‐values from the tests of association between SNPs and phenotype. However, we found that some methods did perform slightly better than the others. In particular, methods that assume a complex genetic architecture of the trait such as SkyNet and SPLS achieved a small positive correlation between the observed and predicted phenotypes. Additionally, methods whose assumptions match the apparent genetic architecture of the trait performed better than methods that do not, as illustrated by the better performance of sparse methods such as the lasso (compared to other methods) on the MTX SJC28 data set.

A minor caveat in the analysis of the MATURA data is that the precise duration of the treatment was not the same for all the subjects in the study; it included 3 and 6 months measures, similar to the anti‐TNF study of Sieberts et al. (2016). However, for the anti‐TNF data, the 6 months measures was available for most of the subjects. Also, for subjects with both 3 and 6 months follow‐up measures, the changes in treatment response were very similar (Supporting Information Figure 5). In view of this fact, and also in accord with Taylor et al. (2018) and Massey et al. (2018), we used the 6 months follow‐up measure, or 3 months if this was not available, noting that for the MTX data set the precise duration of treatment was anyway not available.

We hypothesised that having a strong signal and/or larger sample size would improve the prediction. To investigate this issue, we analysed three additional data sets: (a) a simulated data set of the same size as the anti‐TNF (CRP) data set (1,088 individuals) with a few significant SNP effects (the SimSparse data set), (b) a data set of the same size as the anti‐TNF (CRP) data set where the phenotype was simulated assuming a polygenic architecture (the SimPoly data set), and (c) a much larger real data set with a number of significant SNP effects (the PBC data set). The simulation study showed that the prediction methods were sensitive to violation of the assumptions about the genetic architecture of the trait. In particular, for the sparse data set, sparse methods that were consistent with the data generating mechanism performed the best among all the methods investigated, while for the polygenic data, polygenic methods performed better. On the other hand, methods that were inconsistent with the data generating mechanism generally achieved poor prediction. However, the prediction of the polygenic methods (GCTA‐GREML) improved for the sparse data when the data was reduced to the regions around each true causal SNP. This suggests that prior knowledge of the genetic architecture of the trait, if available, could help to choose the optimal method for prediction (Warren et al., 2014). For the PBC data set, all methods achieved reasonable prediction, although prediction performance was slightly worse than for the simulated data. It is interesting that all the methods performed comparably well for the PBC data set, despite the differences between the methods in terms of sparsity/nonlinearity. This suggests that increasing the sample size may help to overcome the sensitivity of the methods to violation of the assumptions about the genetic architecture of the trait, which is usually unknown.

Our results are relatively consistent with prior work investigating the prediction ability of SNPs derived from genome‐wide association studies of complex traits. Warren et al. (2014) found that the PMSEs achieved when predicting low‐density lipoprotein (LDL) and HDL cholesterol in the Whitehall II and British Women’s Health and Heart Study cohorts barely outperformed the naive prediction method of simply assigning everyone the mean trait value. Spiliopoulou et al. (2015) also considered prediction of HDL (along with height and body mass index) in two data cohorts originating from Croatia and Scotland, and noted that the predictive signal in the genomic data available was still too low for clinical decision‐making at the level of the individual. Several studies (Clayton, 2009; Cleynen et al., 2016; Hamshere et al., 2011; Pashayan et al., 2015; Pharoah, Antoniou, Easton, & Ponder, 2008; Sawcer, Ban, Wason, & Dudbridge, 2010) have shown that use of a limited number of top ranking SNPs can help discriminate diseased cases from unaffected controls, or between different disease sub‐phenotypes, but that the utility for individual risk prediction generally falls far short of clinically useful levels. In some cases this limitation can be overcome by increased sample size at the discovery (model‐building) stage. For example Dudbridge (2013) showed that the disappointing AUCs reported by Machiela et al. (2011) were entirely consistent with the theoretical AUC values of 52–54% predicted on the basis of their discovery set sample size, but that these values could be increased to ≈80−90% if the samples were infinitely large. We reiterate that the maximum achievable AUC will always be limited by the trait heritability. However, even when the combined set of SNPs explain a large proportion of variance, much larger sample sizes are required to achieve high prediction accuracy (Yang et al., 2017) because the individual SNP effects are substantially smaller than the total variance they explain. This could explain why, in the current application, we find genetic predictors alone to have limited predictive value. However other clinical variables or biomarkers (e.g., related to baseline measurements of gene expression or DNA methylation) may provide stronger predictive ability and would be worth further investigation. One of the limitations of this study is that it uses clinical measures of treatment response that do not capture the biology of treatment response (Centola et al., 2013). Clinical measures used as outcome in this study are only moderately correlated with biological measures such as thickness of synovial lining measured by ultrasound scores (Hurnakova et al., 2015). Moving forwards, there is a need for a biological measure of treatment response that is closely reflective of synovitis. Further work that would integrate prediction methods with biological markers of response of synovial tissue might provide better insight into prediction of treatment response. However, this will require investment from partner organisations such as industry and academic partners with access to relevant patient samples.

Our simulation study shows that methods that match the data generating mechanism perform better that the methods that do not. However, for the real data the true genetic architecture is unknown. Therefore, in this study, we applied a variety of prediction methods that cover a wide range of genetic architectures. We note that the list of the methods we applied is by no means exhaustive. Numerous genome‐wide prediction methods have been proposed in the literature including Bayesian approaches (Fragoso, deAndrade, Pereira, Rose, & Soler, 2016; Lee, van der Werf, Hayes, Goddard, & Visscher, 2008; Meuwissen, Hayes, & Goddard, 2001, 2009), dimensionality reduction approaches (Hoggart, Whittaker, DeIorio, & Balding, 2008; Solberg, Sonesson, Woolliams, & Meuwissen, 2009; Wang & Leng, 2016), multiple regression approaches (Ueki & Tamiya, 2016), and others. Nevertheless, the methods explored in this study cover a wide range of genetic architectures in terms of the number and the size of the assumed underlying genetic effects, as well as a variety of methodologies such as sparse, polygenic, machine learning, parametric and nonparametric approaches. We acknowledge that future research may benefit from an exhaustive comparison of the prediction methods available.

## Supporting information

Supplementary Information

Supplementary Information

Supplementary Information

Supplementary Information

Supplementary Information

Supplementary Information

## Lasso (least absolute shrinkage and selection operator)

Lasso (Tibshirani, 1996) is a penalised regression approach that allows shrinkage of the estimators of the regression coefficients in a linear model towards zero using an $L_{1}$ penalty. The $L_{1}$ penalised regression minimises the residual sum of squares subject to the sum of the absolute values of the coefficients being less than a constant. The objective function to minimise is

$${\left(y-X\beta \right)}^{2}+\lambda ||\beta |{|}_{\ell_{1}},$$

where $||\beta |{|}_{\ell_{1}}=\sum_{i=1}^{p}|\beta_{i}|,p$ is a number of the coefficients. Lasso performs variable selection by shrinking some of the coefficient estimates to zero such that only a subset of the coefficients from the lasso fit is used for the prediction. In our analysis, we applied the lasso regression implemented in the R package glmnet (https://cran.r-project.org/web/packages/glmnet).

## Ridge regression

Ridge regression (Cessie & Houwelingen, 1992) allows shrinkage of the estimators of the regression coefficients in a linear model using an $L_{2}$ penalty. The $L_{2}$ penalised regression minimises the residual sum of squares subject to the sum of the squares of the coefficients being less than a constant. The objective function to minimise is

$${\left(y-X\beta \right)}^{2}+\lambda ||\beta |{|}_{\ell_{2}},$$

where $||\beta |{|}_{\ell_{2}}=\sum_{i=1}^{p}\beta_{i}^{2},p$ is a number of the coefficients. Ridge does not allow the shrinkage of the coefficients to exactly zero, and therefore does not perform variable selection. In our analysis, we applied ridge regression implemented in the R package glmnet (https://cran.r-project.org/web/packages/glmnet).

## Elastic net regression

Elastic net (Zou & Hastie, 2005) is a penalised regression that combines both $L_{1}$ and $L_{2}$ penalties. The objective function to minimise is

$${\left(y-X\beta \right)}^{2}+\lambda \left(\frac{1}{2}\left(1-\alpha \right)||\beta |{|}_{\ell_{2}}+\alpha ||\beta |{|}_{\ell_{1}}\right),$$

where $||\beta |{|}_{\ell_{1}}=\sum_{i=1}^{p}|\beta_{i}|$, $||\beta |{|}_{\ell_{2}}=\sum_{i=1}^{p}\beta_{i}^{2}$, p is a number of the coefficients, α is a penalty weight (0<α1). When α=1, the elastic net is identical to lasso, whereas when α=0, it is identical to ridge (Friedman et al., 2010). We used α=0.5. Similarly to lasso, elastic net performs variable selection by shrinking some of the coefficient estimates to zero such that only a subset of the coefficients from the elastic net fit is used for the prediction. In our analysis, we applied elastic net regression implemented in the R package glmnet (https://cran.r-project.org/web/packages/glmnet).

## Sparse partial least squares (SPLS)

SPLS (Chun & Keleş, 2010) is an extension of partial least squares (PLS) that allows for sparsity. PLS is a dimensionality reduction approach, which is based on latent decomposition of the prediction matrix X and the response matrix Y: $Y={\text{TQ}}^{T}+F$, $X={\text{TP}}^{T}+E$, where P and Q are matrices of loadings, F and E are matrices of random errors, and T is a matrix of latent components T=XW, where the columns of $W=\left(w_{1},w_{2},\ldots ,w_{k}\right)$ are direction vectors that capture the most variable directions in the X‐space, and also relate X to Y:

$$w_{k}=\text{arg}\max\left\{{\text{Cor}}^{2}\left(\text{Y, Xw}\right)\times \text{Var}\left(\text{Xw}\right)\right\}.$$

SPLS imposes sparsity at the dimensionality reduction step by specifying the objective function as follows:

$$-kw^{T}\text{Mw}+\left(1-k\right){\left(c-w\right)}^{T}M\left(c-w\right)+\lambda_{1}||c|{|}_{\ell_{1}}+\lambda_{2}||c|{|}_{\ell_{2}},$$

where $M=X^{T}{\text{Y Y}}^{T}X$, c is a surrogate of the direction vector instead of the original vector w, $||c|{|}_{\ell_{1}}$ and $||c|{|}_{\ell_{2}}$ are $L_{1}$ and $L_{2}$ penalties. In our analysis, we applied the SPLS algorithm implemented in the R package spls (http://cran.r-project.org/web/packages/spls).

## Support vector regression (SVR)

In SVR (Vapnik, 1995), the objective function to minimise is:

$$\frac{1}{2}||\beta |{|}^{2}+C\sum_{i=1}^{n}L\left(e_{i}\right),$$

where $||\beta |{|}^{2}=\beta^{T}\beta$, $e_{i}=y_{i}-f\left(x\right)$, $f\left(x\right)=\sum_{i}\beta_{i}k\left(x,x_{i}\right)+b$, b is the bias.

Here, we applied the ϵ‐SVR model which has a sparse solution. In the ϵ‐SVR model, an ϵ‐intensive loss function

$$L_{\epsilon }\left(e\right)=\left\{\begin{array}{cc} 0\; & \text{if}\;|e|<\epsilon \\ |e|-\epsilon \; & \text{otherwise} \end{array}\right.$$

is used, where ϵ is a prespecified threshold. In our analysis, we used a nonlinear kernel, specifically the Gaussian radial basis function (RBF):

$$k\left(x,x_{i}\right)=\text{exp}\left(-||x-x_{i}|{|}^{2}∕\sigma^{2}\right),$$

which allows nonlinear relationships between the SNPs and the phenotype. The method is implemented in the R package mlr (https://cran.r-project.org/web/packages/mlr).

## Random forests (RF)

RF (Breiman, 2001) is a method that generates a collection of regression or classification tree‐structured predictors, or trees. Each tree is a representation of a recursive partitioning of the data set into more and more homogeneous groups, down to terminal nodes. Trees are grown until further partitioning provides less than some minimal amount of extra information.

A random forest is built by repeatedly selecting a number of training data sets with replacement, and growing trees for each data set. Each node on the tree is split using the best (in terms of residual sum of squares) among a subset of predictors randomly chosen for consideration at that node. In a forest of B trees, the random forest predictor is the mean predictor of the individual trees:

$${\hat{f}}_{rf}^{B}\left(x\right)=\frac{1}{B}\sum_{i=1}^{B}T\left(x,\Theta_{b}\right),$$

where $\Theta_{1},\ldots {,\Theta }_{B}$ are independent and identically distributed (i.i.d.) random vectors that define the parameters of the trees in the forest, such as the structure of the trees, which variables are split at which notes, and so on (Hastie et al., 2009). As a nonparametric method, RF allows for interactions and nonlinearity. In our analysis, we used the R package ranger (https://cran.r-project.org/web/packages/ranger).

## Genome‐wide complex trait analysis (GCTA‐GREML)

GCTA‐GREML (Yang et al., 2011) implements linear mixed model analysis, where the effects of the SNPs are assumed to be random effects, with the resulting covariance matrix being proportional to the GRM between individuals. The vector of phenotypes y is represented as

y=Xβ+Wu+ϵ,

where β is a vector of the fixed effects, $u\sim N\left(0,I\sigma_{u}^{2}\right)$ is a vector of the SNP (random) effects, $\epsilon \sim N\left(0,I\sigma_{\epsilon }^{2}\right)$ is a vector of the residuals, and W is a standardised genotype matrix. Defining $\sigma_{g}^{2}=n\sigma_{u}^{2}$ as the variance explained by all SNPs, and defining the matrix A as $A={\text{WW}}^{T}∕n$ (n is the number of SNPs), the vector of phenotypes y can be represented as

y=Xβ+g+ϵ,

where $g\sim N\left(0,A\sigma_{g}^{2}\right)$ is a vector of random genetic effects for the individuals and A is the GRM. This dual formulation of y can be utilised to transform the predicted total genetic effects g of the individuals to SNP effects u. Predicted SNP effects can be then used to predict genetic values in new individuals. We note that although GCTA‐GREML and ridge regression are related methods, they are equivalent under certain conditions only. The best linear unbiased predictor of SNP effects in GCTA‐GREML is equivalent to ridge regression estimator only if the ridge penalty λ =  $\sigma_{\epsilon }^{2}∕\sigma_{\beta }^{2}$ (de Vlaming & Groenen, 2015). However, in our analysis the ridge penalty λ is chosen by cross validation (see Table 1).

## Bayesian sparse linear mixed model (BSLMM)

BSLMM (Zhou et al., 2013) is a hybrid approach that includes both a linear mixed model and a sparse regression model, where sparsity is applied to the fixed effects. The vector of phenotypes y is represented as

$$y=1_{N}\mu +X\beta +u+\epsilon ,$$

where μ is an overall phenotype mean, $1_{N}$ is an N‐vector of 1’s, β is a vector of fixed effects, $u\sim N\left(0,\sigma_{b}^{2}\tau^{-1}K\right)$ is a vector of random effects, $\epsilon \sim N\left(0,\tau^{-1}I\right)$ is a vector of residuals, $K={\mathrm{XX}}^{T}∕n$ is a GRM, n is the number of SNPs and X is a genotype matrix. Fixed effects are assumed to be distributed according to a point‐normal distribution (a mixture of a normal distribution and a point mass at zero):

$$\beta_{i}\;\sim \;\pi N\left(0,\sigma_{a}^{2}\tau^{-1}\right)+\left(1-\pi \right)\delta_{0}.$$

The parameters of the model are μ, τ, π, $\sigma_{a}$, and $\sigma_{b}$, where μ and $\tau^{-1}$ control the phenotypic mean and the residual variance, π controls the proportion of the nonzero fixed effects, $\sigma_{a}$ controls the expected magnitude of the nonzero fixed effects, and $\sigma_{b}$ controls the expected magnitude of the random effects. In practice, the model is re‐parameterised in terms of the expected proportion of the phenotypic variance explained by the sparse fixed effects and the expected proportion of the phenotypic variance explained by the random effects. BLSMM is implemented using Markov chain Monte Carlo (MCMC) techniques to obtain samples from the posterior distribution of the parameters. We used the software implementation included within the GEMMA software package at http://stephenslab.uchicago.edu/software.html.

## SkyNet

SkyNet (Graff et al., 2014) implements an artificial neural network (ANN). The ANN is a computational model based on the collection of nodes that are connected in layers, where the signal travels from the input layer to the output layer, including possible hidden layers. The ANN consists of interconnections between different layers of nodes, the weights of the interconnections, and the activation functions for converting nodes’ weighed input to their output. Input and output layers of the ANN can be described by neural network functions

$$h_{j}=g_{in}\left\{\theta_{j}+\sum_{l}\left(w_{jl}x_{l}\right)\right\}$$

and

$$y_{i}=g_{out}\left\{\theta_{i}+\sum_{j}\left(w_{ij}h_{j}\right)\right\}$$

respectively, where index l represents input nodes, index j represents hidden nodes, index i represents output nodes, w are weights, θ are biases and g is an activation function (Bridges et al., 2011). For hidden layers, the activation function $g\left(z\right)=1∕\left(a+e^{-z}\right)=sig\left(z\right)$ (sigmoid), and for the output layer $g_{out}\left(h\right)=h$. The nonlinearity of the activation function for the hidden layer allows the network to model nonlinear relationships between SNPs and phenotype. In our analysis, we used one hidden layer as recommended by Graff et al. (2014). However, we could not follow the recommendation of using 2n+1 nodes for a hidden layer (n is a number of the input nodes) due to computational constraints. We therefore used seven nodes which is a default number of nodes in the SkyNet software. We investigated the sensitivity of the results to the various other numbers of nodes (up to 100 nodes) and found a very little difference in the predictive performance.

## PRSice

PRSice (Euesden et al., 2015) computes polygenic risk scores (PRS) which are sums of the SNP allele dosages weighted by their estimated effects, with contributing SNPs selected at different *p*‐value thresholds; the final *p*‐value threshold chosen is that which provides the best prediction as assessed via internal cross validation. The PRS is given by

$$PRS=\sum_{i}{\hat{\beta }}_{i}x_{i},$$

where ${\text{x}}_{i}$ is the allelic count at the ith SNP, and ${\hat{\beta }}_{i}$ is the estimated effect at that SNP.

## LDpred

LDpred (Vilhjálmsson et al., 2015) is a polygenic risk score method that accounts for linkage disequilibrium between the SNPs using an external reference panel provided by the user. It estimates the posterior mean of SNP effects for different proportion of true causal SNPs. The SNP effects are assumed to have a Gaussian mixture prior $\beta_{i}\sim N\left(0,h^{2}∕np\right)$ with probability p, and $\beta_{i}=0$ with probability 1−p, where $h^{2}$ is the heritability, n is the total number of SNPs and p is the proportion of the causal SNPs. The posterior mean for the SNP effects is approximated numerically by using an approximate MCMC Gibbs sampler.
