# Supplementary figures and images for: Prediction of treatment response in rheumatoid arthritis patients using genome‐wide SNP data
Source: Genet Epidemiol. 2018 Oct 12;42(8):754–71. doi: 10.1002/gepi.22159 (PMC6334178; doi:10.1002/gepi.22159)

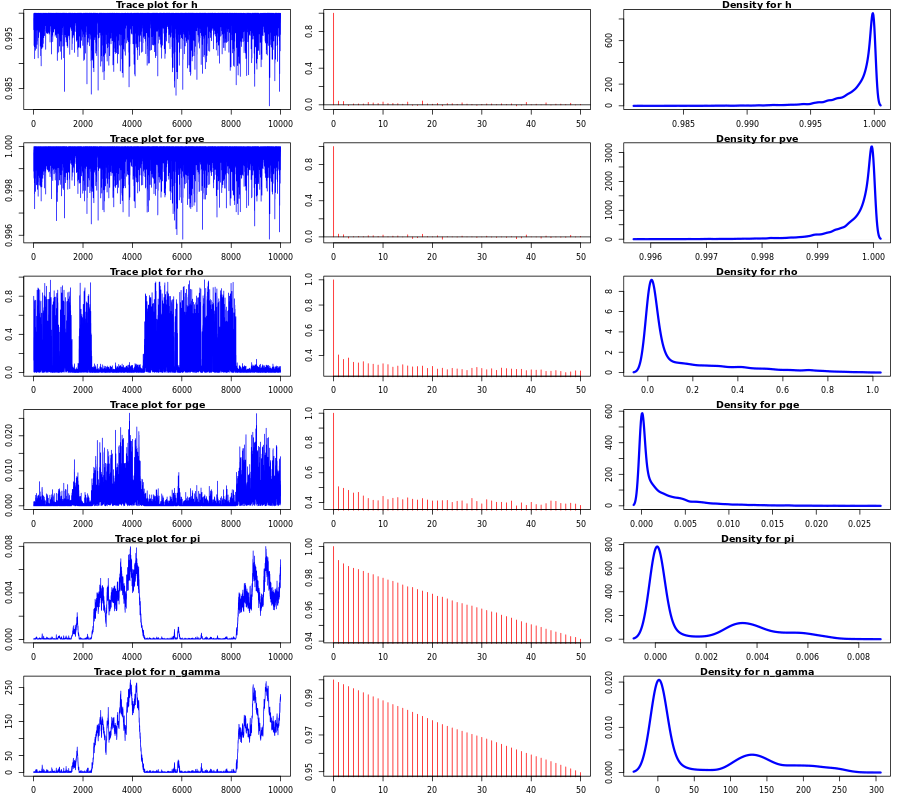

Supplement: Supplementary file 1 — Supplementary Information [file GEPI-42-754-s001.tif]

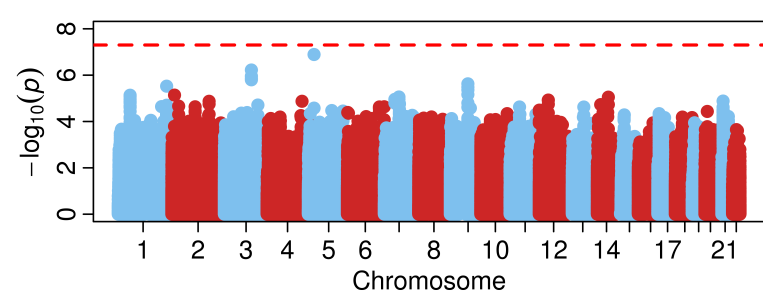

(a)

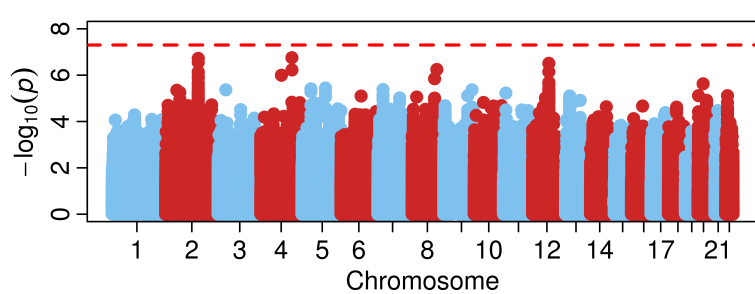

(b)

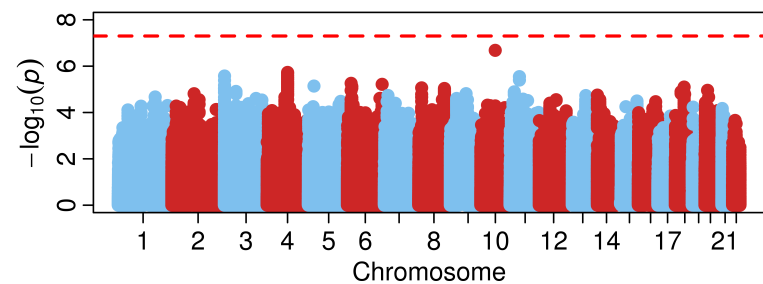

(c)

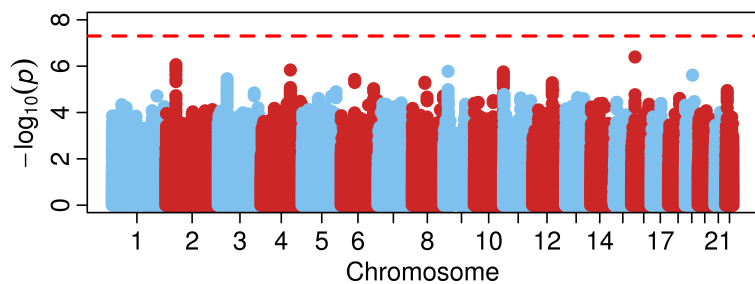

(d)

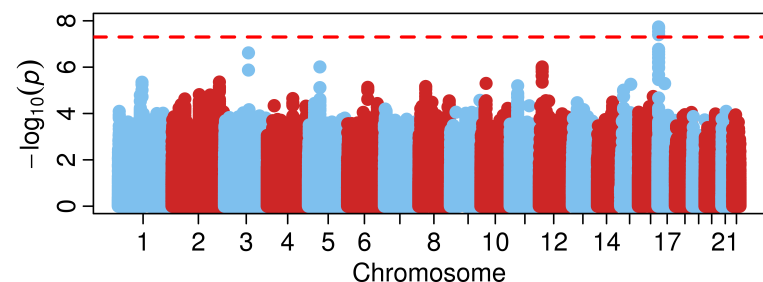

(e)

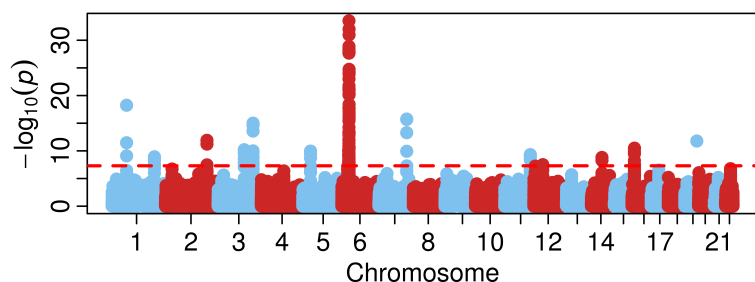

(f)

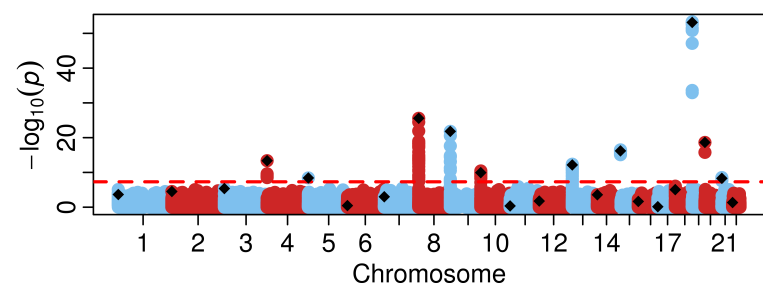

(g)

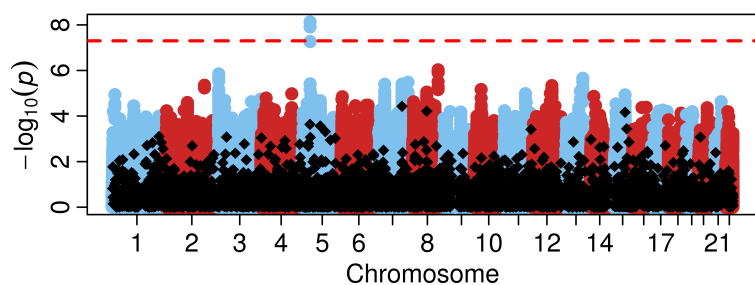

(h)

Supplement: Supplementary file 2 — Supplementary Information [file GEPI-42-754-s002.pdf]

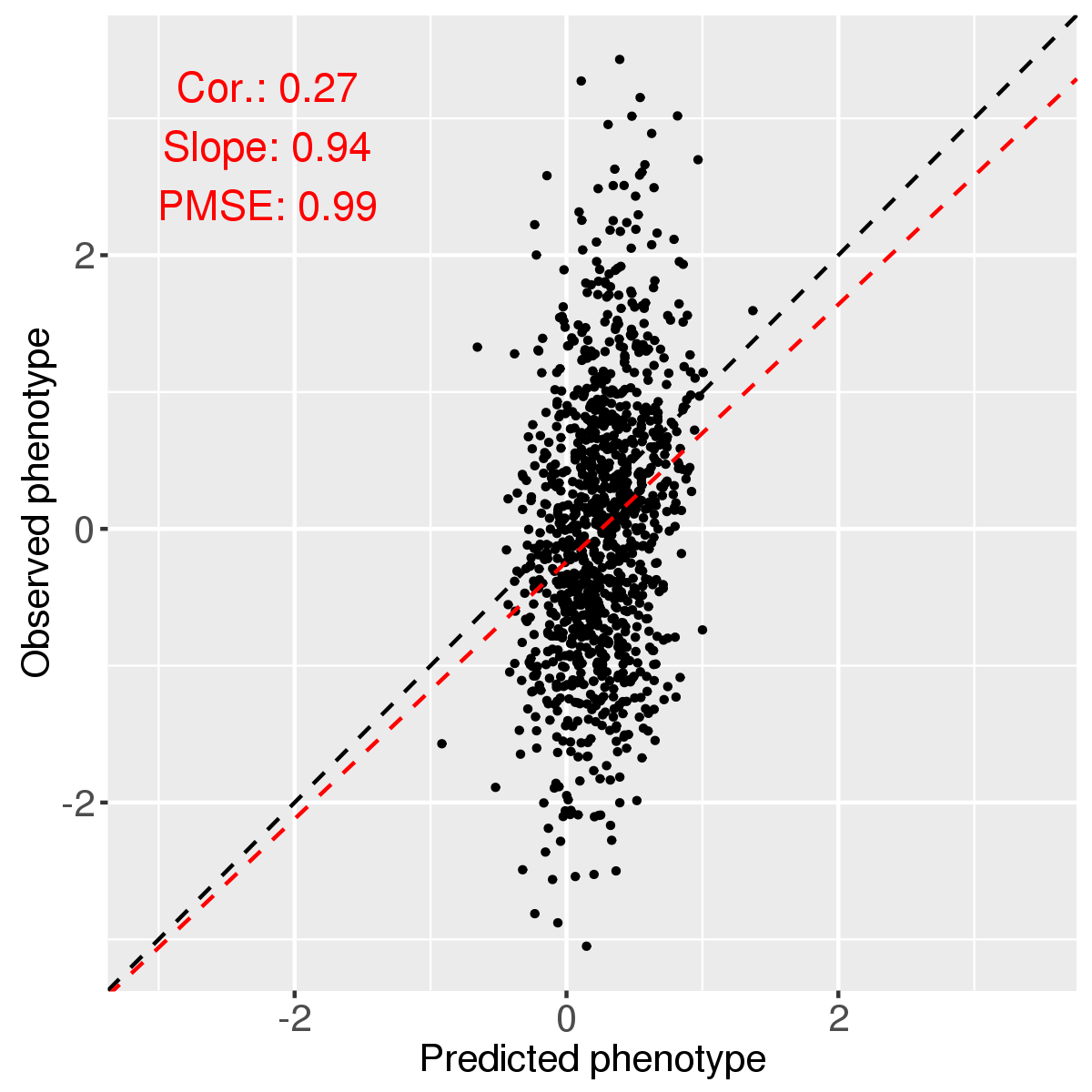

Supplement: Supplementary file 3 — Supplementary Information [file GEPI-42-754-s003.tif]

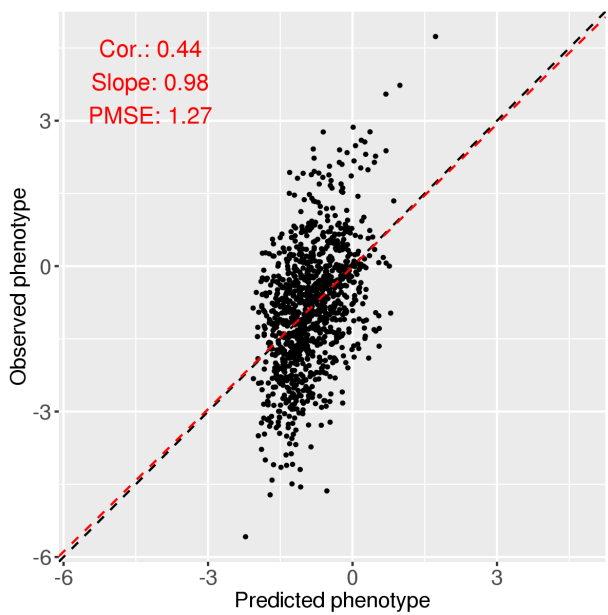

(a)

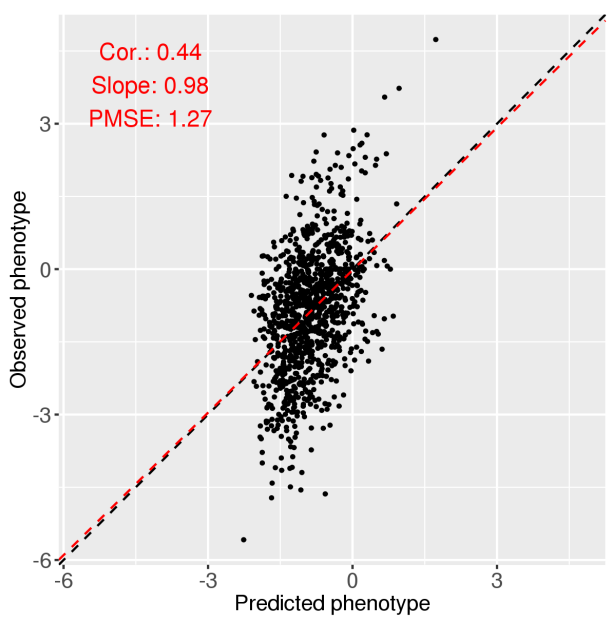

(b)

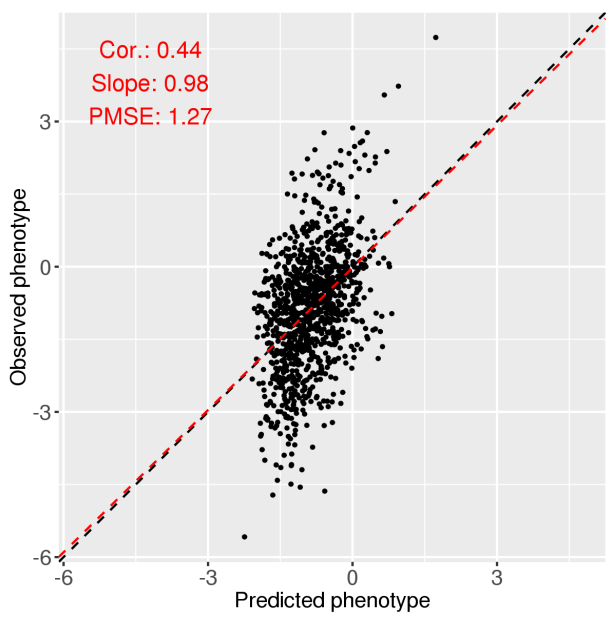

(c)

Supplement: Supplementary file 4 — Supplementary Information [file GEPI-42-754-s004.pdf]

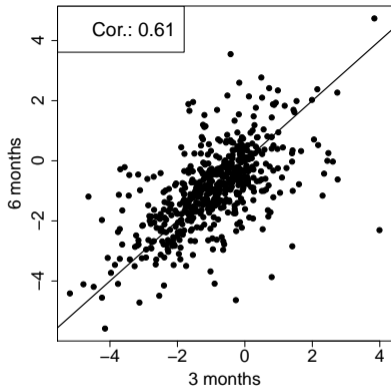

(a) anti-TNF (CRP)

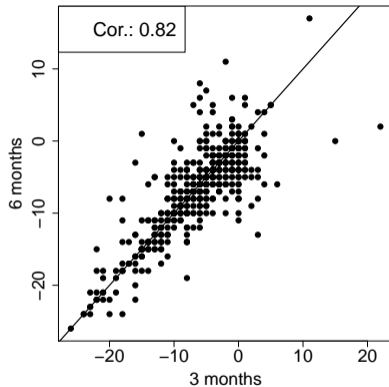

(b) anti-TNF (SJC28)

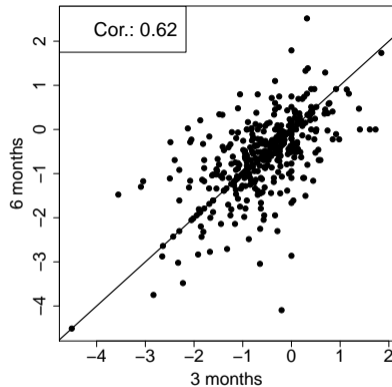

(c) anti-TNF (ESR)

Supplement: Supplementary file 5 — Supplementary Information [file GEPI-42-754-s005.pdf]
