# Supplementary material for: Prediction of treatment response in rheumatoid arthritis patients using genome‐wide SNP data
Source: Genet Epidemiol. 2018 Oct 12;42(8):754–71. doi: 10.1002/gepi.22159 (PMC6334178; doi:10.1002/gepi.22159)
Supplement: Supplementary file 6 — Supplementary Information [file GEPI-42-754-s006.pdf]

Data Set	GCTA-SC Estimated h^2 (SE)	GCTA-MS Estimated h^2 (SE)	GCTA-LD Estimated h^2 (SE)	LDSC Estimated h^2 (SE)	LDAK Estimated h^2 (SE)	Bayesian Posterior mode of h^2 (90% HDI)
Anti-TNF (CRP)	0 (0.247)	0.066 (0.279)	0 (0.288)	0.206 (0.433)	0.024 (0.378)	0.048 (0.001 - 0.313)
Anti-TNF (SJC28)	0 (0.151)	0.09 (0.166)	0.097 (0.186)	0 (0.281)	0.255 (0.232)	0.053 (0.001 - 0.228)
Anti-TNF (ESR)	0.462 (0.173)	0.413 (0.176)	0.647 (0.206)	0.405 (0.326)	0.534 (0.269)	0.466 (0.288 - 0.781)
MTX (CRP)	0 (0.469)	$\star$	$\star$	0.245 (0.891)	0.187 (0.678)	0.091 (0.0001 - 0.571)
MTX (SJC28)	0 (0.45)	$\star$	$\star$	0 (0.886)	0 (0.657)	0.053 (0.00009 - 0.4)
